# Differential Strategies to Tolerate Flooding in *Polygonum hydropiper* Plants Originating From Low- and High-Elevation Habitats

**DOI:** 10.3389/fpls.2018.01970

**Published:** 2019-01-09

**Authors:** Xin-Sheng Chen, Ya-Fang Li, Yun-He Cai, Yong-Hong Xie, Zheng-Miao Deng, Feng Li, Zhi-Yong Hou

**Affiliations:** ^1^Key Laboratory of Agro-ecological Processes in Subtropical Region, The Chinese Academy of Sciences, Changsha, China; ^2^Dongting Lake Station for Wetland Ecosystem Research, Institute of Subtropical Agriculture, The Chinese Academy of Sciences, Changsha, China; ^3^The Faculty of Geography and Resources Sciences, Sichuan Normal University, Chengdu, China

**Keywords:** local adaptation, flooding tolerance, carbohydrate storage, phenotypic plasticity, ecotype differentiation

## Abstract

In species that occur over a wide range of flooding conditions, plant populations may have evolved divergent strategies as a consequence of long-term adaptation to local flooding conditions. In the present study, we investigated the effects of a flooding gradient on the growth and carbohydrate reserves of *Polygonum hydropiper* plants originating from low- and high-elevation habitats in the Dongting Lake wetlands. The results indicated that shoot length did not differ, whereas the total biomass and carbohydrate reserves were reduced under flooded compared to well-drained conditions for plants originating from both habitat types. However, shoot length, shoot mass, rhizome mass, and total biomass were lower in plants from low-elevation habitats than in those from high-elevation habitats in the flooded condition. Soluble sugar and starch contents in belowground biomass were higher in plants from low-elevation habitats than in those from high-elevation habitats independently of the water level. Therefore, *P. hydropiper* plants from low-elevation habitats exhibit a lower growth rate and more conservative energy strategy to cope with flooding in comparison with plants from high-elevation habitats. Differential strategies to cope with flooding among *P. hydropiper* populations are most likely a response to the flooding pressures of the habitat of origin and may potentially drive ecotype differentiation within species along flooding gradients.

## Introduction

Flooding is considered to be a major determining factor in plant growth and species distribution in riparian and lacustrine wetlands (van Eck et al., 2004; Dwire et al., 2006; Luo et al., 2008). Flooding constrains the growth of emergent macrophytes mainly by reducing oxygen availability, ranging from deficiency (hypoxia) to absence (anoxia) of oxygen in highly reduced soils (Vervuren et al., 2003). The strategies of plants to cope with flooding-induced low oxygen can be categorized as the escape strategy and the quiescence strategy (Bailey-Serres and Voesenek, 2008, 2010; Striker et al., 2012). In the escape strategy, the plant grows and/or elongates its shoot to reach the surface and restore contact with the atmosphere (Bailey-Serres and Voesenek, 2008; Manzur et al., 2009). However, the process of shoot elongation is costly under low-oxygen conditions, i.e., the energy required for growing tissues would lead to faster carbohydrate depletion by anaerobic metabolism (Bailey-Serres and Voesenek, 2008, 2010). In contrast, the quiescence strategy is characterized by stable functional traits, such as minimum growth and conservation of carbohydrate reserves (Setter and Laureles, 1996; Sauter, 2000; Bailey-Serres and Voesenek, 2008).

The escape strategy is proposed to be beneficial only in shallow and prolonged floods, where shoot de-submergence is generally possible (Pierik et al., 2009; Chen et al., 2011; Striker et al., 2012). The quiescence strategy is speculated to be beneficial in deep and temporary floods, where shoot emergence might incur higher energy cost and affect eventual recovery when water recedes (Colmer and Voesenek, 2009; Akman et al., 2012; Striker et al., 2012). Along the elevational gradient in wetlands, flood duration and flood depth are highly associated, i.e., plants distributed at high-elevation sites usually experience shallow and temporary flooding, while plants distributed at low-elevation sites often experience deep and long-lasting flooding. This raises the question whether plant populations experiencing deep and prolonged flooding exhibit the quiescent strategy, and plant populations experiencing shallow and temporary flooding use the escape strategy.

Generally, whether plant species exhibit the escape or the quiescence strategy depends on genetics and the flooding regimes in their natural habitats (Akman et al., 2012; Striker et al., 2012). For example, *Rorippa amphibia* (L.) Besser, occupying habitats with long-lasting and relatively shallow floods, adopts the escape strategy and *Rorippa sylvestris* (L.) Besser, occupying habitats with deeper and transient floods, adopts the quiescence strategy (Akman et al., 2012). However, some species occur over a large range of flooding conditions. It is estimated that approximately 30% of the common trees of the Amazonian floodplains also grow in non-flooded upland forests (Ferreira et al., 2009). With long-term adaptation to local flooding conditions, plant populations may have evolved divergent survival and tolerance strategies to cope with distinct flooding regimes (Chen et al., 2009; Ferreira et al., 2009; Mollard et al., 2010). However, few experimental studies have found significant differences in flooding tolerance strategies among populations (Lenssen et al., 2004; Li et al., 2016; Zhang et al., 2016).

In the present study, we investigated the effect of flooding gradients on the growth and carbohydrate reserves of *Polygonum hydropiper* (L.) Delarbre plants originating from two habitats with contrasting flooding conditions in the Dongting Lake wetlands. *Polygonum hydropiper* grows along flooding gradients from low-elevation, lakeside habitats to high-elevation, landside habitats. We hypothesized that *P. hydropiper* from low-elevation sites would utilize the quiescence strategy to survive deep and prolonged flooding, whereas *P. hydropiper* from high-elevation sites would employ the escape strategy to survive shallow and temporary flooding. Hence, morphological traits and the contents of non-structural carbohydrates (i.e., soluble sugars and starch) of *P. hydropiper* plants collected from these two habitat types were evaluated in relation to four water table levels; specifically, drained (water level at -15 cm), waterlogged (0 cm), semi-submergence (15 cm), and complete submergence (30 cm).

## Materials and Methods

### Study Species

*Polygounum hydropiper* (Polygonaceae) is distributed along the sides of streams and riverbanks, and in wet valleys in temperate and subtropical regions of Asia, Australia, Europe, and North America (Li et al., 2003). The branched stems of *P. hydropiper* are normally 40–70 cm long. It is described as an annual herb (Li et al., 2003), but it produces overwintering belowground rhizomes in the Dongting Lake wetlands (Chen et al., 2014, 2015; Pan et al., 2014). *Polygonum hydropiper* is one of the dominant species in the Dongting Lake wetlands and is usually distributed adjacent to the water body and extends to the embankment (Xie and Chen, 2008).

### Experimental Design

The experiment was conducted using wild plants growing in a monitoring plot of the Dongting Lake Station for Wetland Ecosystem Research of the Chinese Academy of Sciences (Yueyang, Hunan, China). On February 10, 2014, plant cuttings of *P. hydropiper* from a low-elevation site (29°27′42.52^′′^N, 112°47′30.09^′′^E, 25.0 m a.s.l.) and a high-elevation site (29°27′54.79^′′^N, 112°46′30.67^′′^E, 29.4 m a.s.l.) along a small-scale elevational gradient in the monitoring plot were collected and transported to the Dongting Lake Station for Wetland Ecosystem Research. Although these two sites differed only by 4.4 m in elevation and were located ∼1.6 km apart, they have important differences in flooding regime. In 2014, the low- and high-elevation sites were submerged during 194 and 71 days, respectively, and mean flooding depths were 3.47 ± 1.84 m (mean ± SD) and 0.98 ± 0.87 m, respectively, at the sampling sites.

Plant cuttings were planted at a depth of approximately 5 cm in a nursery bed containing a soil/sand mixture (1:1 v/v). The soil was collected from the upper layer of the plant collection site and contained 0.19% total nitrogen and 0.08% total phosphorus. After the establishment of plants from the cuttings, on April 6, 80 similar-sized plants (40 plants per habitat type) were selected, planted into individual PVC tubes (height, 30 cm; diameter, 30 cm) that were filled with 30 cm soil, and allowed to grow. On April 20, 48 similar-sized plants (24 plants per habitat type, 28–30 cm in height) were selected for the experiment.

The experimental design was a randomized block with six replicates; the experiment was performed in six outdoor water tanks (100 × 100 × 100 cm) with eight plants (one plant per habitat type per water level) in each tank. The water level in the tanks was maintained at 60 cm (30 cm above the soil surface in the 30-cm water-level treatment). Different water-level treatments within a water tank were established by placing the PVC tubes on either the bottom of the tank (0 cm) or on concrete benches at different heights (15, 30, and 45 cm). The plants placed on the bottom of the tank and on the 15-cm concrete benches were regarded as flooded plants. The plants placed on the 30- and 45-cm concrete benches were regarded as waterlogged (0 cm for the plants) and well-drained (-15 cm for the plants) plants, respectively. Water in the tank was replaced monthly, and filamentous algae were removed manually at regular intervals.

The plants were harvested on August 18, 120 days after the treatment was started, which is more than half of the flooding duration in the low-elevation site. The plants were carefully removed from the tubes to keep the roots and rhizome structures intact. The plants were then cleaned with tap water and transferred to the laboratory for measurements. The branches and rhizomes were counted. Shoot length was calculated as the distance from the shoot base to the tip of the longest leaf. Each plant was then separated into shoots, roots, and rhizomes. The biomass of each plant part was measured after drying in an oven at 80°C for 48 h. Total biomass was determined as the total plant dry weight at the end of the experiment.

The contents of starch and soluble sugar were analyzed using a modification of the method of Yemm and Willis (1954). The dry belowground biomass including roots and rhizomes of each plant was ground to a fine powder and extracted three times using 80% ethanol (v/v). Then, 0.5 ml of anthrone reagent and 5 ml oil of vitriol were added to the extract. The mixture was heated for 10 min in boiling water and then cooled quickly in an ice bath, followed by measurement of the absorbance at 630 nm using a spectrophotometer (Shimadzu, Kyoto, Japan). The residue remaining after soluble sugars extracted was dried, extracted with 30% perchloric acid, and analyzed for starch (as glucose equivalent) by spectrophotometry using the anthrone reagent.

### Statistical Analyses

The significance of differences in total biomass, shoot length, numbers of branches and rhizomes, and of the contents of soluble sugar and starch of plants from the two habitat types under different water-level treatments was assessed using general linear models, with water level and habitat type included as main factors and block as a random factor (McKone and Lively, 1993). We further conducted linear contrasts to test the effect of water level in each plant habitat type, and the effect of plant habitat type at each water level. The least significant difference (LSD) method was used to compare means among the four water-level treatments (Sokal and Rohlf, 1981). Data were log_10_-transformed if necessary to reduce the heterogeneity of variances, and heterogeneity was confirmed using Levene’s test. All statistical analyses were performed using SPSS 15.0 (SPSS Inc., Chicago, IL, United States).

## Results

### Plant Biomass

**FIGURE 1:**
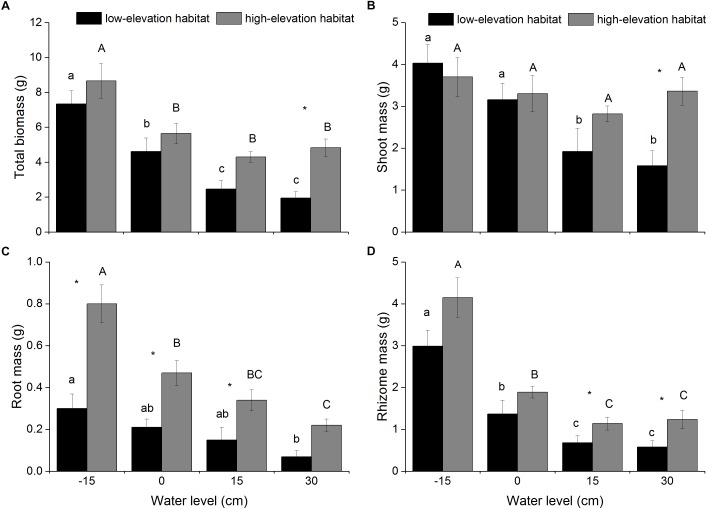
Total biomass **(A)**, shoot mass **(B)**, root mass **(C)**, and rhizome mass **(D)** of *P. hydropiper* plants originating from two habitats grown under four water levels. Different lower-case or capital letters indicate that means differ significantly between the water-level treatments for plants originating from low-elevation and high-elevation habitats, respectively. Symbols (^∗^) show which means differed between habitat types at each of the four water levels.

Total biomass, shoot mass, and rhizome mass produced by *P. hydropiper* were significantly affected by habitat type and water level (Table 1). The total biomass and rhizome mass of *P. hydropiper* plants were significantly lower in the flooding treatments (15- and 30-cm water levels) than in the well-drained treatment (-15-cm water level) (Figures 1A,D). The shoot mass was significantly lower in the flooding treatments than in the well-drained treatment for plants from the low-elevation habitat, but not for plants from the high-elevation habitat (Figure 1B). In the 30-cm flooding treatment, plants from the high-elevation habitat produced higher shoot mass, rhizome mass, and total biomass than those from the low-elevation habitat (Figures 1A,B,D).

**Table 1:** Summary of two-way analysis of variance (*F*-values) for shoot mass, root mass, rhizome mass, and total biomass; shoot length; branch and rhizome numbers; and the contents of soluble sugar and starch in *P. hydropiper* from two habitat types under four water levels.

Effect	Total biomass	Shoot mass	Root mass	Rhizome mass	Shoot length	Number of branches	Number of rhizomes	Starch content	Water-soluble sugar
Habitat type (H)	11.94^∗∗^	4.29^∗^	37.47^∗∗∗^	17.33^∗∗∗^	18.35^∗∗∗^	0.22^ns^	0.09^ns^	68.03^∗∗∗^	137.91^∗∗∗^
Water level (N)	18.78^∗∗∗^	5.60^∗∗^	14.35^∗∗∗^	35.56^∗∗^	0.45^ns^	2.54^ns^	3.74^∗^	10.23^∗∗∗^	14.39^∗∗∗^
H × N	0.56^ns^	2.13^ns^	3.00^∗^	0.22^ns^	1.28^ns^	0.51^ns^	0.13^ns^	2.46^ns^	0.95^ns^


*^∗∗∗^P < 0.001; ^∗∗^P < 0.01; ^∗^P < 0.05; ^ns^P > 0.05.*

Root mass produced by *P. hydropiper* was significantly affected by habitat type and water level, with significant interactions between both factors (Table 1). Root mass was significantly lower in the 30-cm flooding treatment than that in the well-drained treatment (Figure 1C). Root mass was higher in plants from the high-elevation habitat than in those from the low-elevation habitat, except at 30-cm water level (Figure 1C).

### Shoot Length, Number of Branches, and Rhizomes

**FIGURE 2:**
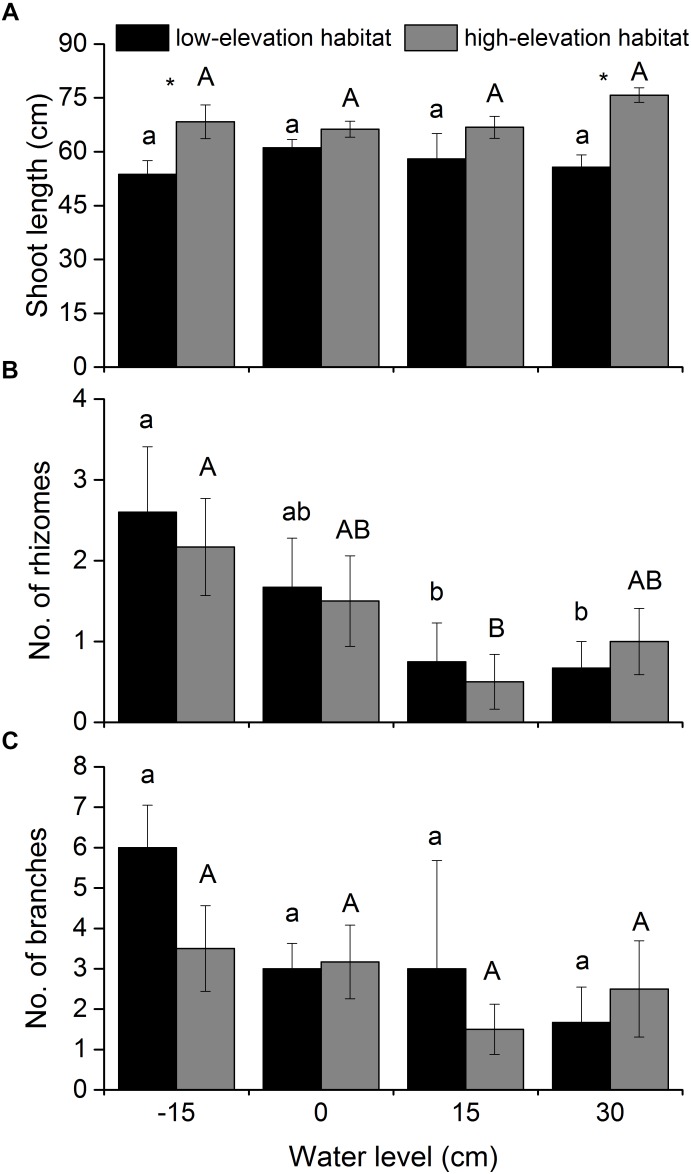
Shoot length **(A)**, branch number **(B)**, and rhizome number **(C)** of *P. hydropiper* plants originating from two habitats grown under four water levels. Different lower-case or capital letters indicate that means differ significantly between the water level treatments for plants originating from low-elevation and high-elevation habitats, respectively. Symbols (^∗^) show which means differed between habitat types at each of the four water levels.

Shoot length of *P. hydropiper* plants was only significantly affected by habitat type (Table 1 and Figure 2A). In the -15- and 30-cm water-level treatments, shoot length was higher in plants from the high-elevation habitat than in those from the low-elevation habitat. The number of rhizomes produced by *P. hydropiper* was significantly affected by water level (Table 1 and Figure 2B). *Polygonum hydropiper* plants in the 15-cm flooding treatment produced less rhizomes than plants in the well-drained (-15-cm water level) condition. The number of branches produced by *P. hydropiper* was neither affected by water level nor by habitat type (Table 1 and Figure 2C).

### Non-structural Carbohydrate Contents

**FIGURE 3:**
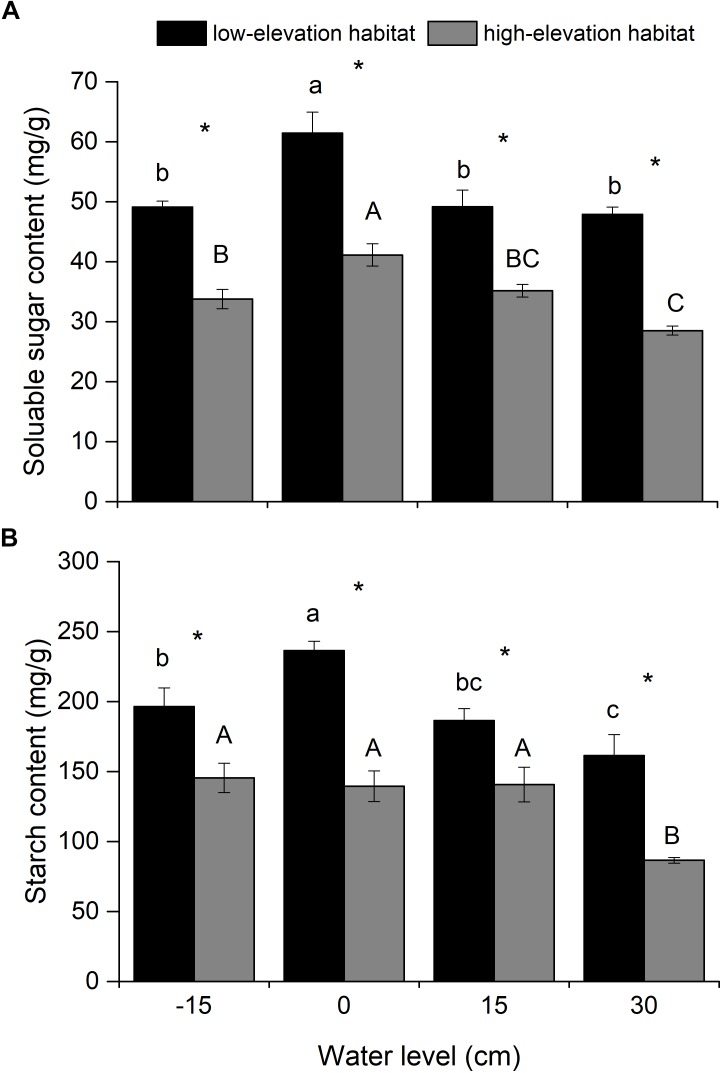
Soluble sugar **(A)** and starch **(B)** contents in belowground biomass (roots and rhizomes) of *P. hydropiper* plants originating from two habitats grown under four water levels. Different lower-case or capital letters indicate that means differ significantly between the water level treatments for plants originating from low-elevation and high-elevation habitats, respectively. Symbols (^∗^) show which means differed between habitat types at each of the four water levels.

Soluble sugar and starch contents were significantly affected by habitat type and water level (Table 1). The soluble sugar content was lower in the flooding treatments (15- and 30-cm water levels) than in the waterlogged condition (0-cm water level; Figure 3A). The starch content was lower in the 30-cm flooding treatment than in the well-drained and waterlogged treatments (-15- and 0-cm water level; Figure 3B). Soluble sugar and starch contents of plants from the low-elevation habitat were higher than those in plants from the high-elevation habitat at all water levels (Figures 3A,B).

## Discussion

Shoot length did not differ among flooding treatments for plants either from low-elevation or high-elevation habitats, indicating that *P. hydropiper* did not exhibit an apparent escape strategy to cope with flooding. Shoot elongation is considered an efficient approach for escaping submergence stress, and a key attribute in a low-oxygen escape strategy (Chen et al., 2009). However, elongation growth competes with maintenance processes for carbon used for survival during complete submergence (Ram et al., 2002). Moreover, if air contact cannot be established, high carbohydrate consumption will lead to an energy deficit, severe tissue damage, and mortality (Pierik et al., 2009; Chen et al., 2011). It was found that elongation growth by leaves is negatively correlated with seedling survival under complete submergence in rainfed lowland rice cultivars (Ram et al., 2002). In our flooding treatments, shoots of *P. hydropiper* emerged from the water. However, in their habitats of origin, flooding was deep and long-lasting even for *P. hydropiper* populations in the high-elevation habitat (mean flood depth 0.98 m and 71 days). Elongation growth may not be sufficient for shoots of *P. hydropiper* to regain contact with the air, and therefore the escape strategy was not selected.

Flooding generally reduced the total biomass and carbohydrate reserve of *P. hydropiper* from both high-elevation and low-elevation habitats. Emergent macrophytes like *P. hydropiper* have evolved a range of morphological and physiological adaptations, such as stem or petiole elongation, narrower and thinner leaves, short and thick roots, a shallow root system, aerenchyma formation, glycolysis, and fermentation to acquire oxygen or diminish root oxygen demand in flooded environments (Chen et al., 2009; Xie et al., 2009; Pan et al., 2014). However, emergent plants usually lack the ability to sustain photosynthetic activity when submerged in water (Macek et al., 2006). Extended periods of anoxic conditions may eventually result in the reduction of plant growth and total biomass (Edwards et al., 2003).

Although flooding generally reduced the growth of *P. hydropiper* plants from both habitat types, shoot length and total biomass in the 30-cm flooding treatment were lower in plants from the low-elevation habitat than those from the high-elevation habitat. These results indicated that *P. hydropiper* plants from the low-elevation habitat exhibit a smaller modular size and lower growth rate under complete submergence than plants from the high-elevation habitat. Previous studies have also found a reduced modular size in lakeside genotypes in comparison with landside genotypes of *Ranunculus reptans* L. (Lenssen et al., 2004). During submergence, a low growth rate and small modular size may result in lower respiratory losses and consequently, in high carbohydrate reserves (Lenssen et al., 2004). Consistent with this hypothesis, water-soluble sugar and starch contents were higher in *P. hydropiper* plants from the low-elevation habitat than in those from the high-elevation habitat. A high carbohydrate supply is related to flooding tolerance under complete submergence (Ram et al., 2002; Qin et al., 2013). Large carbohydrate reserves may enable *P. hydropiper* plants to tolerate deep and long-lasting flooding in low-elevation habitats. Therefore, differences in growth and carbohydrate reserves under complete submergence between plants from low- and high-elevation habitats are most likely a response to the flooding pressures of the habitat of origin.

Flooding can act as a strong selective pressure for key adaptations, potentially leading to ecotype differentiation or even speciation (Lenssen and de Kroon, 2005; Voesenek et al., 2006). Mollard et al. (2010) have shown that subtle topographical differences along a floodplain may promote different plant strategies among *Paspalum dilatatum* Poir. populations and drive within-species ecotype differentiation. Lenssen et al. (2004) reported that flooding can induce genetic differentiation in the clonal species *R. reptans* along a small-scale gradient. In the present study, *P. hydropiper* populations from habitats differing in flooding regimes exhibited different morphological and physiological responses to flooding, suggesting there is a potential for population differentiation along flooding gradients (Chen et al., 2009; Ferreira et al., 2009).

## Conclusion

Our results indicated that flooding reduced growth and carbohydrate reserves of *P. hydropiper* plants originating from both high-elevation and low-elevation habitats. However, low-elevation and high-elevation populations of *P. hydropiper* respond differently to flooding disturbances. Under complete submergence, the low-elevation population showed a smaller modular size and lower growth rate in comparison with the high-elevation population. Soluble sugar and starch contents were higher in plants from the low-elevation habitat than in those from the high-elevation habitat independently of the water level. Therefore, *P. hydropiper* plants from the low-elevation habitat exhibit a lower growth rate and more conservative energy strategy to cope with flooding in comparison with plants from the high-elevation habitat. These different adaptive strategies to tolerate flooding among *P. hydropiper* populations may potentially drive ecotype differentiation within this species along flooding gradients.

## Author Contributions

X-SC and Y-FL wrote the manuscript and executed the technical assays and statistical analyses. X-SC and Y-HX designed the experiments and edited the manuscript. Y-HC, Z-YH, Z-MD, and FL contributed to data collection and interpretation. All authors reviewed the manuscript.

## Conflict of Interest Statement

The authors declare that the research was conducted in the absence of any commercial or financial relationships that could be construed as a potential conflict of interest.
